# A case report: Acute fibrinous and organizing pneumonia

**DOI:** 10.1097/MD.0000000000036093

**Published:** 2023-11-24

**Authors:** Chao Liu, Wei Chen, Yongjun Deng, Siqi Li, Yulin Liu, Jianping Liang

**Affiliations:** a Department of Respiratory and Critical Care Medicine, Zhongshan City People Hospital, Zhongshan, Guangdong Province, China.

**Keywords:** acute fibrinous and organizing pneumonia, interstitial lung diseases, lung biopsy

## Abstract

**Rationale:**

Acute fibrinous and organizing pneumonia (AFOP) is a rare acute or subacute interstitial lung disorder characterized by the deposition of fibrin within the alveoli and organizing pneumonia with a patchy distribution. The clinical features of AFOP are nonspecific, and it is often misdiagnosed as pneumonia, cancer, tuberculosis, or other lung disorders.

**Patient concerns:**

In this case report, a 58-year-old woman presented with chest tightness, shortness of breath, cough and sputum. A chest CT scan showed multiple patchy shadows in both lungs. She was initially diagnosed with community-acquired pneumonia. Her purified protein derivative skin test was positive, but sputum was negative for acid-fast bacilli.

**Diagnoses:**

AFOP was diagnosed by bronchoscopic lung biopsy and histopathology.

**Interventions:**

Following AFOP diagnosis, all anti-infective drugs were discontinued, and replaced by methylprednisolone and prednisone.

**Outcomes:**

After 1 week of treatment with methylprednisolone 40 mg daily, the patient chest CT and clinical symptoms improved. After 1 month, the patient symptoms had demonstrated dramatic improvement and CT scan revealed complete absorption of lesions in both lungs. After 5 months of follow-up, the patient symptoms completely disappeared.

**Lessons:**

Acute AFOP is an uncommon lung condition with poor prognosis; hence, early diagnosis and identification are particularly important. Definitive diagnosis requires histopathological findings. Currently, there is no unified treatment guideline for AFOP, and treatment must be tailored based on the etiology and severity of each individual patient disease. Subacute AFOP shows a good response to corticosteroid treatment.

## 1. Introduction

Acute fibrinous and organizing pneumonia (AFOP) is an uncommon lung disease with an unusual pattern of interstitial pneumonia. AFOP is characterized by the deposition of fibrin “balls” within the alveoli and organizing pneumonia (OP) distribution.^[1]^ AFOP diagnosis is often delayed, mainly due to nonspecific clinical symptoms and radiographic findings. At present, definitive diagnosis is obtained from lung biopsy and histopathology. Effective drug treatment is achievable using corticosteroids. Herein, we present a case report of a middle-aged woman presenting for the first time with chest tightness, and who was diagnosed with AFOP through bronchoscopic lung biopsy. The patient eventually recovered under steroid treatment.

## 2. Case report

A-58-year-old female was transferred to our department with a 5-week history of chest tightness. At a local hospital, her chest X-ray scan demonstrated right lower lung consolidation. She was treated with cefuroxime, but her symptoms did not improve. Later, she went to the outpatient clinic of our hospital and received a 1-week course of anti-infective agents (cefaclor and moxifloxacin). However, her symptoms were still not significantly alleviated. She had a history of shingles and denied smoking and drinking. She denied any recent travel, exposure to any chemicals, or any pets. She denied any history of hypertension, diabetes, heart disease, liver disease, or kidney disease.

**Figure 1.**
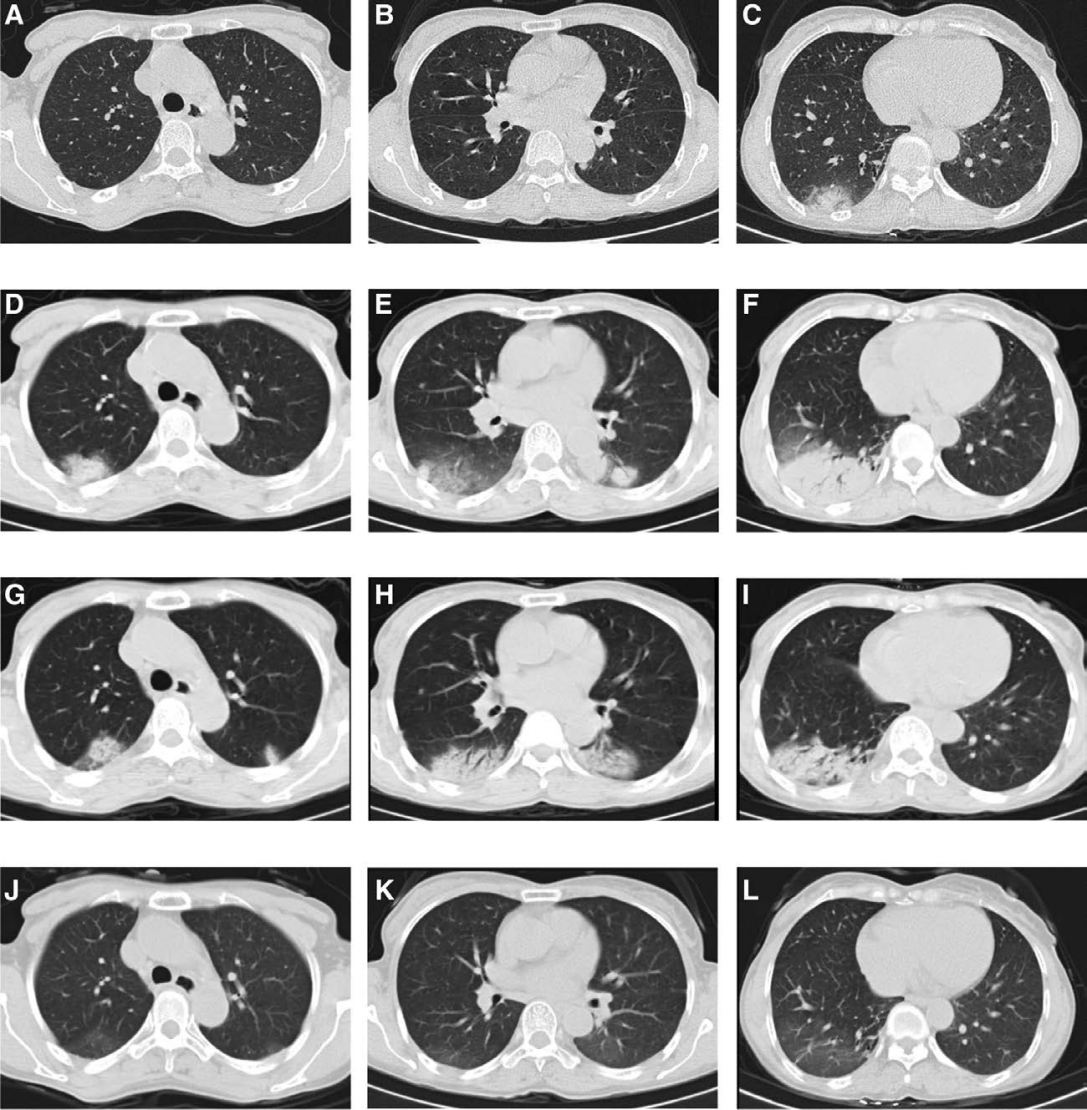
The initial and follow-up chest CT images of this case report. High resolution CT images at admission showed consolidation in the right lower lung (A–C). CT images at the 7th day of admission showed a significant increase in the patchy infiltrates in both lungs (D–F). CT images at the 8th day of the steroid treatment showed that the patchy infiltrates in both lungs had shrunken (G–I). CT images after 1 month revealed complete absorption of bilateral lung lesions (J–L).

On initial examination, her vital signs were as follows: temperature 36.7°C; pulse rate 100/min; respiratory rate 20 breaths/min; blood pressure 122/78 mm Hg; oxygen saturation with room air 95%. Chest auscultation revealed increased breath sounds on both sides with no dry and wet rales. All her other physical examination results were normal. Laboratory data showed a white blood cell count of 7.12*10^9/L, hemoglobin of 114.0 g/L, platelet count of 199*10^9/L, ESR of 120 mm/h, C-reactive protein of 75.46 mg/L, serum procalcitonin of 0.056 ng/mL, D-dimer of 0.53 mg/L, and albumin of 34.8 g/L. Purified protein derivative (PPD) skin test results were strongly positive, but three consecutive sputum samples for acid-fast bacilli, sputum smears, and sputum cultures were negative. The patient’s autoimmune antibody profile (anti-nuclear antibodies, anti-dsDNA antibody, anti-neutrophil cytoplasmic antibody, rheumatoid factor), tumor biomarkers (CEA, NSE, CYFRA 211, CA 125), N-terminal brain natriuretic peptide, serum troponin T, and 1-3-β-D Glucan were within normal limits. No obvious abnormalities were found on ECG or cardiac color ultrasound. High resolution chest CT scan showed consolidation in the right lower lung (Fig. 1A–C).

Piperacillin tazobactam sodium was administered initially as anti-infective therapy, but there was no significant improvement in clinical symptoms. One week following the commencement of antibiotic treatment, chest CT (Fig. 1D–F) examination showed that the consolidation lesions in the lower lobe of the right lung occupied greater area than previously, and new lesions were found in the upper and lower lobes of the left lung. On the 8th day of admission, the patient developed shortness of breath following physical activity, accompanied by cough and white mucus sputum. At the same time, piperacillin tazobactam sodium was switched to moxifloxacin for anti-infection treatment. Subsequently, a CT-guided percutaneous needle lung biopsy was performed, and histological examination demonstrated widened alveolar septa, a large amount of exudate in the alveolar cavities, and a small amount of lymphocyte and eosinophilic infiltration.

**Figure 2.**
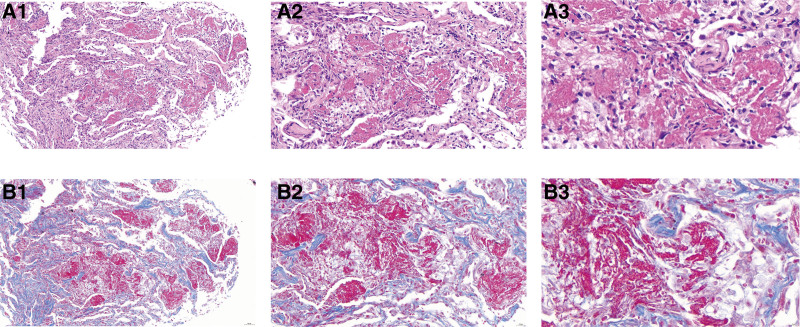
Histopathology of lung tissue sample collected by bronchoscopy. Hematoxylin and eosin (H&E) staining (A1, 100X) (A2, 200X) (A3, 400X) showed multiple “fibrin balls” with organizing in alveolar spaces, fibroblast and lymphocytes infiltration, without hyaline membrane formation. Masson trichrome stain (B1, 100X) (B2, 200X) (B3, 400X) showed alveolar cavities containing multiple fibrinous exudation and fibroblast foci formation.

Due to the strong positive PPD skin test but negative acid-fast bacilli in the sputum, we considered the possibility of tuberculosis. Consequently, bronchoscopy was performed on day 14. The patient bronchoalveolar lavage fluid culture was negative. Histopathology (lower lobe dorsal segment and outer posterior basal segment of the right lung) demonstrated thickened alveolar spaces, scattered fibroblast and lymphocytes infiltration, and massive fibrinous exudate with organization in the alveolar cavities (Fig. 2). Taken together, the patient clinical manifestations and histopathology results were consistent with AFOP.

Once the diagnosis of AFOP was confirmed, antibiotics were discontinued and the patient was treated with intravenous corticosteroids (methylprednisolone, 40 mg per day for 1 week). Her clinical symptoms (chest tightness, dyspnea, cough, and expectoration) were slightly relieved. On the 8th day of steroid treatment, the patient chest CT examination (Fig. 1G–I) showed obvious absorption of the consolidation in both lungs, compared to previously. The patient was discharged with prednisone 25 mg daily. After 1 month, the patient symptoms had shown dramatic improvement and pulmonary CT scan revealed complete absorption of bilateral lung lesions (Fig. 1J–L); therefore, the dose of prednisone was reduced to 10 mg daily. Her condition was basically stable after 3 months and the dose was adjusted to 5 mg per day. At her 5-month follow up, her clinical symptoms had completely disappeared and oral prednisolone was discontinued.

## 3. Discussion

AFOP was first defined by Beasley^[1]^ in 2002 as a rare pattern of response to acute lung injury. There is a sexual dimorphism in incidence, with men more commonly affected by AFOP than women. AFOP is considered a subtype of idiopathic interstitial lung disease and distinct from traditional interstitial lung diseases such as diffuse alveolar injury (DAD), bronchiolitis obliterans with OP, and eosinophilic pneumonia (EP).^[2]^ The etiology of AFOP is complex and may be related to diverse factors such as infection, environmental causes, medications, autoimmune diseases, hematological malignancies, and organ transplantation.^[3,4]^ However, an idiopathic variety of AFOP has previously been reported.^[5–7]^ For the patient described in the present case report, there was no evidence of any associated infection, drugs, environmental exposure, or autoimmune disease; hence, this may be a case of idiopathic AFOP.

AFOP has been reported to be of 2 forms: acute or subacute lung injury. The acute form of lung injury presents with severe respiratory failure and even acute respiratory distress syndrome, leading to poor prognosis and a very high mortality rate. In contrast, the subacute form has better prognosis and a lower mortality rate after drug intervention.^[1,8]^ The clinical manifestations of AFOP show no obvious specificity, and the patients range from infants to the elderly.^[9]^ Common clinical symptoms of AFOP include cough, dyspnea, and fever. Some patients also have chest pain, fatigue, hemoptysis, night sweats, and weight loss.^[10]^ The radiological manifestations of AFOP are insufficient for diagnosis and may vary. The common imaging manifestations of idiopathic AFOP include bilateral diffuse lung consolidation shadows, mainly distributed in the peripheral and basal segments of the lung. However, the most common imaging findings of secondary AFOP are ground-glass opacity, pulmonary nodules, and pleural effusion.^[11]^ Some patients with AFOP may also demonstrate halo sign and reversed halo sign in pulmonary CT.^[6,12]^ The average time from the onset of symptoms to diagnosis has been reported to be 19 days.^[1]^ Most cases are initially misdiagnosed as pneumonia, and only a few cases have been misdiagnosed as lung cancer or other pulmonary disorders. In this patient, the right lung first presented with a patchy solid shadow, followed by multiple patchy solid shadows in the left lung lobe, mainly distributed in the periphery and the lower parts of both lungs. Bronchodilation and thickening of the bronchial wall were found at the site of the consolidation, consistent with previously reported imaging changes in idiopathic AFOP.

In our case report, the patient presented with symptoms associated with community-acquired pneumonia. Inflammatory markers such as CRP and NEUT% were elevated. The symptoms were not relieved after empirical anti-infective treatment, and the lung lesions progressed significantly. Given the similarities between previously reported AFOP cases and our patient clinical manifestations, imaging results, and laboratory tests, we were prompted to prepare to perform a lung biopsy to confirm the diagnosis. Generally, lung tissue specimens can be obtained surgically, or by bronchoscopy, EBUS-GS guidance, CT-guidance, or ultrasound-guidance.^[11,13]^ However, the patient condition should be fully evaluated and the location of the lung lesion confirmed before the most appropriate biopsy method is selected. In the current case report, CT-guided percutaneous lung biopsy was first performed to obtain lung tissue samples, and the pathological diagnosis was considered OP. As the patient was strongly positive for PPD, we decided to re-biopsy the lung via bronchoscopy to rule out tuberculosis, and finally diagnosed AFOP by histopathology of the right lower lung tissue. Therefore, for pneumonia patients with poor outcomes following anti-infection treatment, the possibility of AFOP needs to be considered in differential diagnosis.

The main pathological features of AFOP are the deposition of fibrin “balls” in the alveoli and the distribution of OP.^[1,7]^ However, AFOP may also manifest as a widening of the alveolar space, with no typical transparent membrane formation in the alveoli, no obvious eosinophilic infiltration, and no granuloma or necrotic tissue formation.^[1,14]^ AFOP should be distinguished from diffuse alveolar injury (DAD), cryptogenic OP, and EP, which may exhibit similar histopathological findings. For instance, the histological features of EP include the mixed aggregation of fibrin, eosinophilic cells, and macrophages in the alveoli, and the formation of eosinophilic micro-abscesses in the lung interstitium.^[15]^ The histopathological features of cryptogenic OP are characterized by significant proliferation of fibrous tissue and fibroblasts in the respiratory bronchioles, alveolar ducts, and alveoli, forming typical Masson bodies. However, there is usually almost no fibrin deposition in the alveoli.^[16]^ The histological characteristics of DAD include the infiltration of inflammatory cells and the formation of a diffuse “eosinophilic transparent membrane” in the alveoli, observable under microscopy.^[17]^

At present, there are no unified standard treatment guidelines for patients with AFOP. The treatment guidelines and durations should be determined based on the patient clinical course and etiology. It has been reported that antibiotics, corticosteroids, immunosuppressants (mycophenolate, azathioprine, and cyclophosphamide) and mechanical ventilation may be considered for the treatment of AFOP.^[7]^ At present, glucocorticoids are the most widely used therapeutic drugs for AFOP, but no consensus has been reached on the dosage and course of treatment.^[13,18]^ High-dose pulse therapy or medium-dose corticosteroid maintenance therapy may be suitable for the treatment of AFOP, but patients may relapse when corticosteroid doses are reduced, requiring a higher dose to be re-implemented to alleviate symptoms once more.^[6]^ Bhatti et al^[19]^ reported 1 case of AFOP that was successfully treated by corticosteroid combined with mycophenolate mofetil. Successful treatment of other cases of AFOP have also been reported following corticosteroid combined with ciclosporin or cyclophosphamide.^[20,21]^ Renaud-Picard et al^[22]^ reported a young patient with pulmonary cystic fibrosis who received bilateral lung transplantation and developed AFOP 42 months later. The patient chose to undergo a second bilateral lung transplantation, and his condition was stable after 2 years of follow-up. Interestingly, etanercept, a TNF inhibitor, can also be used as a treatment for AFOP.^[23]^ Another case involved a middle-aged female patient infected with HIV, who developed acute respiratory distress syndrome 11 days after taking abacavir and was ultimately diagnosed with AFOP by histopathology. After discontinuing the medication for 3 days, the patient condition and chest CT showed significant improvement.^[24]^ Therefore, drug discontinuation may be an effective intervention for the treatment of AFOP. In addition, surgical resection is also a treatment option for local lesions in the lungs.^[25]^

Hence, the variety of available treatments and the lack of standardized guidelines illustrate the necessity of thorough consideration of all options for individual patients. For patients with secondary AFOP, especially those with autoimmune diseases, greater benefit may be derived from immunosuppressive drugs. For well-defined lung lesions in AFOP, drug management may not be necessary and surgical intervention may be available. For severe or end-stage AFOP patients, lung transplantation may be an additional treatment option. The patient in the current case report was successfully treated with a medium dose of corticosteroid. This treatment was successful, as the patient lung CT examination showed complete absorption of lesions in both lungs after 1 month, and clinical symptoms disappeared after 5 months of follow-up. The patient disease did not recur during the corticosteroid dose reduction process, but the long-term efficacy of the treatment and possibility of recurrence still require further follow-up observation.

## 4. Conclusion

In conclusion, it remains controversial whether AFOP, as a special interstitial pneumonia, is an independent disease entity. Evidently, the study of AFOP has not attracted enough attention from clinicians. The diagnosis of AFOP is challenging and AFOP should be considered in the differential diagnosis of patients with suspected community-acquired pneumonia who have failed to respond to antibiotic therapy. AFOP has unique histopathological changes, but lacks specific clinical symptoms and imaging findings, so selecting an appropriate lung biopsy method to obtain lung tissues samples is key to a definitive diagnosis. For patients with the acute form of AFOP, prognosis is poor and many patients even require mechanical ventilation treatment. For patients with subacute AFOP, most respond well to corticosteroid therapy, and other treatment options should be selected appropriately according to the etiology and severity of the patient disease.

## Author contributions

**Conceptualization:** Chao Liu, Jianping Liang.

**Data curation:** Jianping Liang.

**Formal analysis:** Chao Liu.

**Investigation:** Wei Chen, Siqi Li.

**Methodology:** Yongjun Deng.

**Software:** Siqi Li, Yulin Liu.

**Writing – original draft:** Chao Liu, Jianping Liang.

**Writing – review & editing:** Chao Liu, Jianping Liang.
